# Proteomic Analysis of Invasive Breast Cancer Cells Treated with CBD Reveals Proteins Associated with the Reversal of Their Epithelial-Mesenchymal Transition Induced by IL-1β

**DOI:** 10.3390/ijms26104721

**Published:** 2025-05-15

**Authors:** Lázaro García-Morales, Emmanuel Ríos-Castro, José Tapia Ramírez, Isaura Meza

**Affiliations:** 1 Department of Molecular Biomedicine, Centro de Investigación y de Estudios Avanzados del Instituto Politécnico Nacional, Avenida Instituto Politécnico Nacional 2508, Ciudad de México 07360, Mexico; lazaro.garcia@cinvestav.mx; 2 Unidad de Genómica, Proteómica y Metabolómica (UGPM), Laboratorio Nacional de Servicios Experimentales (LaNSE), Centro de Investigación y de Estudios Avanzados, Avenida Instituto Politécnico Nacional 2508, Ciudad de México 07360, Mexico; eriosc@cinvestav.mx; 3 Department of Genetics and Molecular Biology, Centro de Investigación y de Estudios Avanzados del Instituto Politécnico Nacional, Avenida Instituto Politécnico Nacional 2508, Ciudad de México 07360, Mexico; jtapia@cinvestav.mx

**Keywords:** cannabidiol, phenotype reversion, protein regulation, proteomics, mass spectrometry, protein networks, cancer treatment

## Abstract

Cannabidiol (CBD) has shown promise in treating cancers with an inflammatory microenvironment. Although it has been demonstrated that IL-1β induces epithelial-mesenchymal transition (EMT) of MCF-7 cells and CBD reverts this process, in restoring the epithelial non-invasive phenotype, there is limited understanding of how this cannabinoid regulates these processes. In this work, MCF-7 cells were induced to adopt an aggressive phenotype (6D cells), which was reversed by CBD. Then, protein expression was analyzed by mass spectrometry to compare 6D vs. MCF-7 cells and 6D+CBD vs. 6D cells proteomes. Novel proteins associated with EMT and CBD signaling were identified. Twenty-four of them were oppositely regulated by IL-1β and CBD, suggesting new points of crosstalk between the IL-1β and CBD signaling pathways. From the data, two protein networks were constructed: one related to EMT with 58 up-regulated proteins and another with 21 related to CBD signaling. The first one showed the proteins BRCA1, MSN, and CORO1A as the key axis that contributes to the establishment of a mesenchymal phenotype. In the CBD signaling, the key axis was formed by SUPT16H, SETD2, and H2BC12, which suggests epigenetic regulation by CBD in the restoration of an epithelial phenotype of breast cancer cells, providing new targets for anticancer therapy.

## 1. Introduction

The epithelial-mesenchymal transition (EMT) is a crucial process leading to cancer progression, invasion, and metastasis, which can be regulated by microenvironmental signals such as hypoxia, inflammation, and epigenetics [1]. EMT is considered a reversible process that involves the loss of epithelial characteristics such as cell-cell junctions, adhesion, and cellular polarity, but the acquisition of invasive properties [1,2]. The inflammatory cytokines TNF-*α*, TGF-β, IL-6, IL-8, and IL-1β have been used as inflammatory stimuli that foster a tumor microenvironment inducing EMT in several cell lines [1,3]. However, it is not yet fully understood how these inflammatory molecules regulate this transition.

Our previous work has shown that IL-1β can induce an EMT process in non-invasive human breast cancer MCF-7 cells, leading to various genotypic and phenotypic changes. The activation of the IL-1β/IL-1R/β-catenin signaling pathway is the underlying mechanism behind the observed changes, which contribute to the transition to an aggressive phenotype in these cells (now referred to as 6D cells) [3,4,5,6,7].

Considering the possibility of reversing EMT through a mesenchymal-epithelial transition (MET), one plausible approach was to block the IL-1β signaling pathway using an anti-inflammatory molecule such as cannabidiol (CBD). It was demonstrated that some phenotypic changes, as well as alterations in the expression of several mRNAs and proteins, induced by IL-1β, were blocked and reversed by CBD [7,8]. These changes included alterations in gene expression related to malignancy, restoration of cell-cell contacts with a clear re-establishment of adherens junctions, an in vivo inhibition of tumor progression, potentially through the induction of a pro-apoptotic process and angiogenesis suppression [7,8].

CBD has been associated with additional anticancer properties and is involved in various signaling pathways that mediate its effects [9]. A proteomic approach has reported mitochondrial dysfunction and inducement of endoplasmic reticulum stress when cancerous cells are treated with this cannabinoid [10]. In addition, CBD can suppress angiogenesis and stem cell-like properties of breast cancer through Src/VHL/HIF-1α signaling [11]. CBD also inhibits the release of exosomes and microvesicles (critical structures that favor inflammation and tumor spread) in a dose-dependent way in some cancer cell types and sensitizes cancer cells to chemotherapy [12]. The activity of CBD has shown lower potency in non-cancer cells compared to other cannabinoids such as tetrahydrocannabinol, cannabigerol, cannabichromene, and cannabidiolic acid [13]. Previously, we reported a similar finding, as CBD exhibited lower activity in MCF-7 cells than in malignant 6D cells [8].

In MCF-7 cells, using a label-free quantification method, synergistic interactions were demonstrated between CBD and chemotherapeutic drugs enhancing the apoptotic effect of these drugs through new signaling pathways which involved the inhibition of topoisomerase II, cullin 1, V-type proton ATPase, and CDK-6 as an additional cytotoxic mechanism of CBD in cancerous cells [14]. Furthermore, in MDA-MB-231 breast cancer cells, CBD and doxorubicin inhibited the methylation of histone H3 at lysine K4 and the acetylation of histone H2 at lysine K5, leading to chemo-sensitization [15]. Previously, we have demonstrated a synergic effect of CBD with cisplatin in both MCF-7 and 6D cells [8]. However, all these new findings also reveal additional properties of CBD, highlighting its capacity to utilize different signaling pathways to induce molecular changes in cancerous cells. CBD can affect the regulation of several physiologic processes, including autophagy, cytokine secretion, apoptosis, and innate and adaptive immune responses. It has been reported that these CBD effects can be inconsistent because of differences in the biological models, cell types, and CBD concentration used [16,17]. All this information makes clear that the anti-inflammatory and antitumoral signaling mechanisms of CBD are not yet fully understood.

Therefore, the aim of this work was to identify other mechanisms, in addition to the one that we have previously reported [3], which contribute to the transition of MCF-7 cells to an aggressive 6D cell phenotype induced by IL-1β. Furthermore, we also searched for new processes that CBD activates to counteract and reverse the transition. Using a proteomic approach, we found new proteins and new crosstalk points between signaling pathways. Through protein-protein interaction analysis, we identified two main functional protein-protein interaction networks that participate in the IL-1β or CBD signaling.

## 2. Results

**Figure 1:**
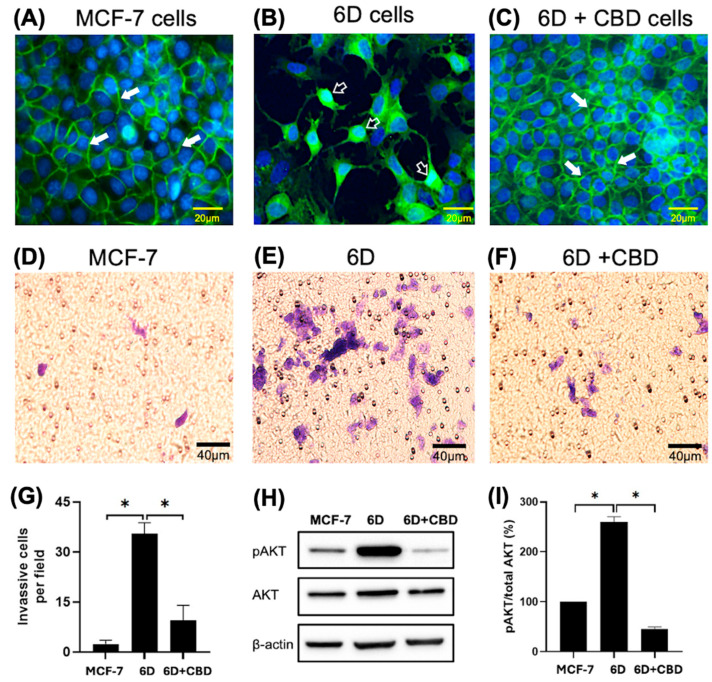
Changes in cell morphology and β-catenin expression in breast cancer cells induced by IL-1β or CBD stimulation. (**A**) Representative image of cell morphology and β-catenin distribution in the control MCF-7, (**B**) 6D, and (**C**) 6D+CBD cells, β-catenin localization in the adherens junctions (full arrows) or in the nuclei (empty arrows) is indicated in each panel. Bar = 20 µm. (**D**) Representative image of MCF-7 cell invasion through Matrigel-coated Transwell inserts. (**E**) 6D and (**F**) 6D+CBD cells. The cells were stained with Giemsa and examined under a light microscope. Five fields per insert were analyzed (Bar = 40 µm). (**G**) Number of cells that invaded Matrigel-coated inserts is represented as mean ± SD of the obtained values, asterisks indicate significance at *p* ≤ 0.05. (**H**) Representative western blot showing AKT and pAKT (Ser 473) levels in MCF-7, 6D, and 6D+CBD cells. (**I**) Relative expression of the percentage of AKT phosphorylation (expressed as pAKT/Total AKT ratio) in each cell type, asterisks indicate significance at *p* ≤ 0.05.

The phenotype and β-catenin distribution of the cells used were confirmed before each experiment by immunofluorescence. Figure 1A shows the presence of β-catenin at the cell-cell contact sites forming adherens junctions in the control MCF-7 cells, which exhibited an epithelial phenotype. In contrast, the malignant 6D cells displayed a mesenchymal phenotype, characterized by a clear loss of cell-cell contacts and the translocation of β-catenin to the nuclei (Figure 1B). When 6D cells were treated with CBD, the epithelial phenotype was reestablished, and β-catenin was localized again at the adherens junctions (Figure 1C). Furthermore, Figure 1D shows the invasiveness of the MCF-7 cells, which was very low when it was evaluated by Matrigel-coated inserts (mean of 2.4 cells per field). In contrast, 6D cells were significantly more invasive than the control cells (mean of 35.4 cells per field) (Figure 1E,G). When 6D cells were treated with CBD, their invasive properties decreased notably (mean of 9.4 cells per field) (Figure 1F,G). In addition, phosphorylation levels of AKT, a key effector in the IL-1β/IL-1RI/β-catenin pathway, were quantified. As we have reported, AKT serves as a crucial crosstalk point between IL-1β and CBD signaling, resulting in phenotype reversion of 6D cells [8]. Our present results confirmed that AKT is highly phosphorylated in 6D cells (Figure 1H). The levels of the phosphorylated protein increased to 260% in 6D cells compared to control MCF-7 cells. While in 6D cells treated with CBD, the levels of pAKT decreased to 45% (Figure 1I).

### 2.1. Differential Protein Expression

**Figure 2:**
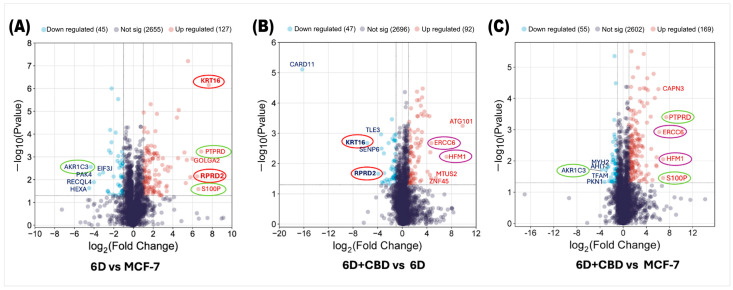
Volcano plots of differential protein expression in the MCF-7, 6D, and 6D+CBD cells obtained by mass spectrometry. (**A**) Distribution of all differentially expressed proteins in the 6D cells vs. control MCF-7 cells. (**B**) Differential protein distribution in the 6D+CBD vs. 6D cells. (**C**) Differential protein distribution in the 6D+CBD vs. control MCF-7 cells. In all graphs, up-regulated proteins (log2FC ≥ 1) are shown as red points and down-regulated (log2FC ≤ −1) ones as blue points, proteins with not-significative changes in the expression are shown as purple points. Data are displayed according to the log2Fold change (horizontal axis) and −log10 of the *p*-value (vertical axis). The black-dotted line on the vertical axis indicates the limits for significance (−log10Pvalue ≤ 0.05). The top five up-regulated and down-regulated proteins are indicated according to their gene symbols. KRT16 and RPRD2, circled in red, were up-regulated by IL-1β and down-regulated by CBD. PTPRD, S100P, and AKR1C3, circled in green, exhibited similar expression patterns in both, 6D vs. MCF-7 and 6D+CBD vs. MCF-7 comparisons. The CBD up-regulated proteins ERCC6 and HFM1 are indicated with purple circles.

Analysis of the proteomes of control MCF-7, 6D, and 6D+CBD cells by mass spectrometry identified changes in protein expression induced by IL-1β or CBD. First, we compared the proteomes of 6D cells with their parental MCF-7 cells to examine the effects of IL-1β and then evaluated the impact of CBD by comparing the proteomes of 6D+CBD cells with those of 6D cells (not treated with CBD) and MCF-7 cells (control). In the 6D vs. MCF-7 comparison, 2827 proteins were shared between these cell types, of which 127 (4.49%) were significantly up-regulated and 45 (1.59%) were down-regulated in cells treated with IL-1β (Figure 2A). Additionally, IL-1β exposure induced the expression of 5 proteins and repressed 2 proteins. As shown in Table 1, the proteins only detected after IL-1β stimulus have different roles including: (a) transcriptional regulation (ZNF347), (b) nuclear transport (IPO9), (c) NAD+ metabolism (SARM1), (d) microtubule remodeling (MTUS2) and (e) cell differentiation (TSNAXIP1). The two proteins, GIPC2 and KLHDC7B, are also shown in Table 1 as being present in control MCF-7 cells but absent in 6D cells, suggesting that they are repressed by IL-1β.

As shown in Figure 2B, by comparing the proteins expressed in the 6D+CBD cells vs. 6D cells to evaluate the effect of CBD on 6D cells, 2835 proteins were shared between them. Of these, 92 (3.24%) were significantly up-regulated by CBD, while 47 (1.65%) were significantly down-regulated. Notably, Figure 2A,B show that KRT16 and RPRD2 are two proteins strongly up-regulated by IL-1β and subsequently down-regulated by CBD, which indicates two points of crosstalk between IL-1β and CBD signaling. Table 2 shows that CBD induced the expression of 12 proteins and repressed 7 proteins.

The KLHDC7B protein was identified to be expressed in 6D+CBD cells (Table 2). This protein, as mentioned above, was absent in 6D cells (Table 1). Therefore, it is uniquely expressed in MCF-7 cells. This implies that KLHDC7B, such as KRT16 and RPRD2, could represent another point of crosstalk between IL-1β and CBD signaling pathways, as CBD restores its expression.

The other CBD-induced proteins (Table 2) has been associated with several functions such as: (a) cell growth, proliferation, and migration (KRT17), (b) transcriptional regulation (PTMA), (c) folate metabolism (DHFR2), (d) translation regulation (RACK1), (e) lipid transport (ABCB4), (f) selenium metabolism (SCLY), (g) microtubule assembly, lamellipodia formation, cell adhesion, migration, and chemotaxis (FER), (h) GTPase activity (ARFGAP1); (i) RNA binding (SRP19); (j) hydrolase activity (DNPEP) and (k) vesicle-mediated transport (COG5) (described in the UNIPROT database [49]).

On the other hand, the proteins repressed by CBD treatment of malignant 6D cells were involved in various processes, including: (a) tumorigenesis by stabilization of p53/TP53 (BRI3BP), (b) serving as structural components of the cytoskeleton (ACTG1), (c) cellular migration (SLIT1), (d) controlling cellular growth (CLIC3), (e) as components of the Golgi apparatus (GOLGA2), (f) RNA binding (HNRNPUL2) and (g) remodeling the cytoskeleton (CRMP1) (Table 2).

By comparing the protein expression between 6D+CBD and control MCF-7 cells, both of which exhibit an epithelial cell morphology (Figure 1A,C), it was found that 2826 proteins were shared between these two cell types, with 169 proteins up-regulated and 55 proteins down-regulated by CBD (Figure 2C). Interestingly, the expression levels of the proteins PTPRD, S100P, and AKR1C3 remained similar to those observed in the comparison between 6D vs. MCF-7 cells, suggesting that some proteins regulated by IL-1β may persist even after CBD treatment.

### 2.2. Pathway Enrichment and Gene Ontology Analyses of Differentially Regulated Proteins

**Figure 3:**
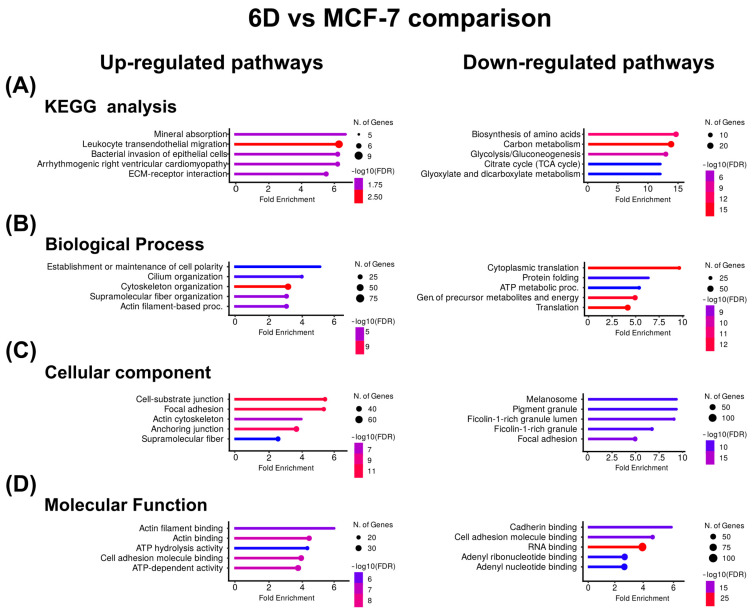
KEGG and Gene Ontology analyses of the proteins with co-expressed patterns identified in the 6D vs. MCF-7 comparison. (**A**) KEGG pathways identified in the up-regulated and down-regulated proteins. (**B**) Pathways associated with biological processes. (**C**) Pathways associated with cellular component. (**D**) Pathways associated with molecular function. In each case, only the five pathways with the highest enrichment are shown.

Based on the clustering results, the KEGG pathway enrichment and gene ontology (GO) analyses were used to identify biologically overrepresented pathways and to examine the functions of the clustered proteins across three main ontologies. It was revealed that both IL-1β and CBD affected a wide range of cellular processes, functions, and components. Figure 3A shows the KEGG pathway enrichment analysis for the proteins up-regulated by IL-1β, indicating associations with mineral absorption and leukocyte transendothelial migration. GO analysis identified the top pathways related to the establishment of cell polarity and cilium organization for biological processes (Figure 3B), cell-substrate junctions, focal adhesion for cellular components (Figure 3C), and actin filament and actin binding for molecular function (Figure 3D).

In contrast, in the proteins down-regulated by IL-1β, KEGG analysis showed that the main down-regulated proteins were related to amino acid biosynthesis and carbon metabolism. Gene ontology indicates that cytoplasmic translation and protein folding were down-regulated processes, melanosome and pigment granule were down-regulated components, and cadherin binding and cell adhesion were down-regulated functions (Figure 3). Table 3 shows the Top ten of up- or down-regulated proteins in the 6D vs. MCF-7 comparison, respectively. Here, the most up-regulated protein is keratin 16 (KRT16), while the most down-regulated is β-hexosaminidase (HEXA).

**Figure 4:**
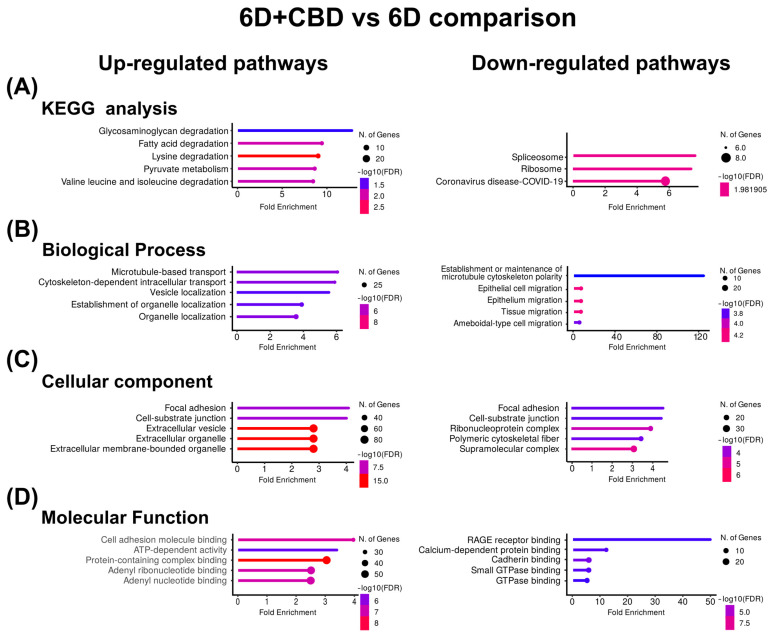
KEGG and Gene Ontology analyses of the proteins with co-expressed patterns identified in the 6D+CBD vs. 6D comparison. (**A**) KEGG pathways identified. (**B**) Pathways associated with biological processes. (**C**) Pathways associated with cellular component. (**D**) Pathways associated with molecular function. In each case, only the five pathways with the highest enrichment are shown.

In the 6D+CBD vs. 6D cells comparison, the KEGG enrichment of the CBD up-regulated proteins reveals that these proteins were associated with the pathways of degradation of glycosaminoglycan and fatty acids (Figure 4A). GO identified the pathways of microtubule-based transport and cytoskeleton-dependent intracellular transport for biological processes, focal adhesion and cell-substrate junction for cellular component, and cell adhesion, molecular binding, and ATP-dependent activity for molecular function (Figure 4B–D). Although in the CBD, down-regulated proteins, KEGG and GO, analyses indicated spliceosome and ribosome for KEGG, establishment or maintenance of microtubule cytoskeleton polarity and epithelial cell migration for biological process (Figure 4B), focal adhesion and cell-substrate junction for cellular component (Figure 4C), and RAGE receptor binding and calcium-dependent protein binding for molecular function (Figure 4D). As shown in Table 4, the most up-regulated protein after CBD treatment is the autophagy-related protein 101 (ATG101), while the most down-regulated is the caspase recruitment domain-containing protein 11 (CARD11).

Analyzing the proteins with opposite effects on their expression levels caused by the IL-1β stimulus or CBD treatment, it was found that another 15 proteins had a similar expression behavior such as KRT16 and RPRD2, as they were up-regulated by IL-1β and then down-regulated by CBD (Table 5). In contrast, we also identified five proteins (KCNH5, HERC4, MYO1D, EMILIN1, EBF3) that were down-regulated by IL-1β and then up-regulated by CBD (Table 5).

### 2.3. Protein-Protein Interactions

Having identified the proteins that are regulated by treatment with IL-1β or CBD, we further explored the complexity of the protein interactions in 6D cells. We investigated whether the up-regulated or down-regulated proteins could form networks, using known and predicted protein-protein interactions reported in the STRING database.

**Figure 5:**
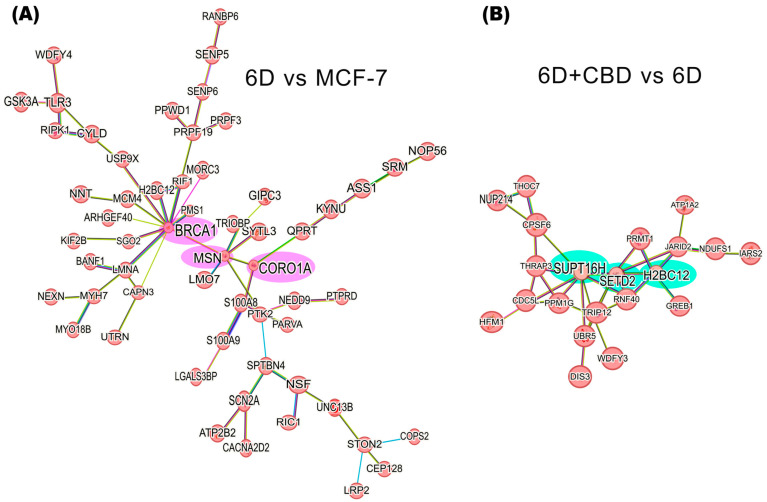
Protein-protein interaction networks of IL-1β or CBD up-regulated proteins in the malignant 6D cells. (**A**) The main network of IL-1β up-regulated proteins in the 6D vs. control MCF-7 cells comparison shows a key axis formed by the proteins BRCA1, MSN, and CORO1A (highlighted in pink). (**B**) The main network of CBD up-regulated proteins in the 6D+CBD vs. 6D comparison shows a key axis formed by the proteins SUPT16H, SETD2, and H2BC12 (highlighted in green). The interaction analyses were performed using STRING, considering only the statistically significant up-regulated proteins (log2FC ≥ 1).

Figure 5A shows the interaction network formed by 58 of the 127 up-regulated proteins in the 6D vs. MCF-7 cells comparison. Based on the predicted interactions, the network encloses three main nodes (hubs), which include the BRCA1, MSN, and CORO1A proteins. In contrast to down-regulated proteins in the same comparison, two small interaction networks were identified. The first includes the proteins RECQL4, STAU1, and ZC3HAV1, and the second involves EIF3J, PRPF4B, and RRP12 (Supplementary Figure S1A).

In the 6D+CBD vs. 6D comparison, the CBD up-regulated proteins reveal a network comprising 21 proteins, with SUPT16H, SETD2, and H2BC12 as the main nodes (hubs) (Figure 5B). At this point, H2BC12 was up-regulated in the 6D cells, and subsequently its expression was increased by CBD, suggesting a potential additive effect of both compounds on its expression. Among the down-regulated proteins in the 6D+CBD vs. 6D comparison, two networks were identified. The first network, associated with neutrophil aggregation, involves the proteins S100A8, ITGAM, S100A9, ANXA1, TLR3, and IFI16. And the second network includes OPTN, GRM5, TUBB3, and RRM1 proteins (Supplementary Figure S1B).

**Figure 6:**
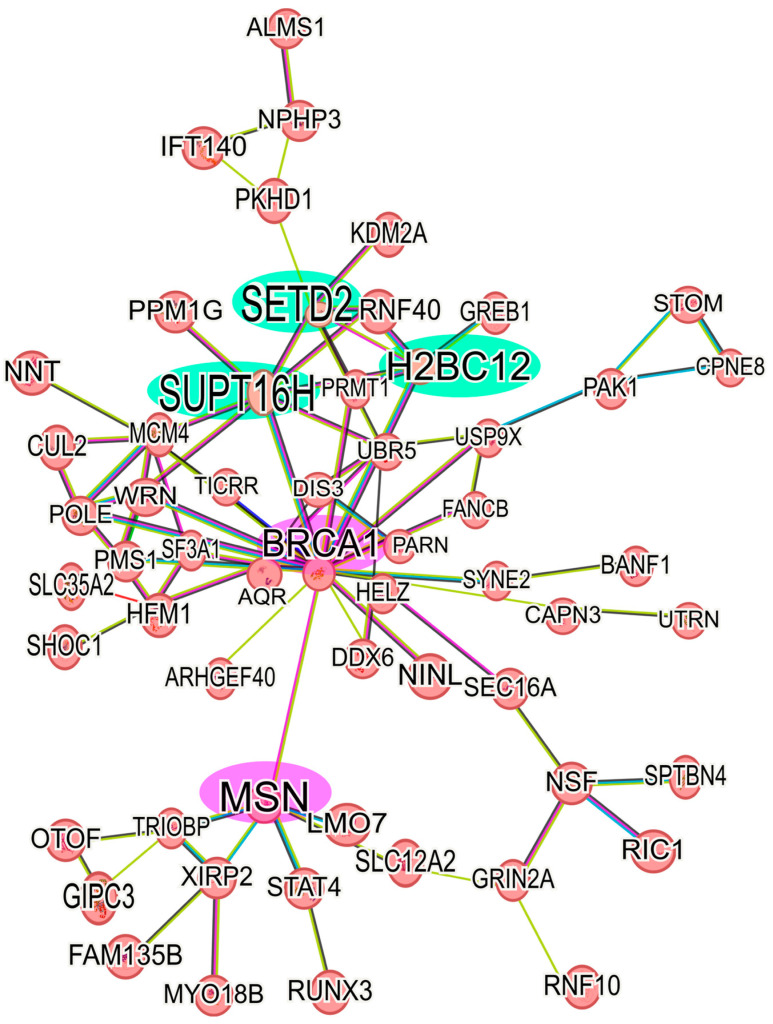
Protein-protein interaction network of up-regulated proteins in 6D+CBD vs. MCF-7 cells. The main network of CBD up-regulated proteins with a log_2_FC ≥ 1 was formed with 58 proteins. The interaction of the key axis formed by the proteins SUPT16H, SETD2, and H2BC12 (highlighted in green) with BRCA1 and MSN (highlighted in pink) is shown. This interaction analysis was performed using STRING, considering only the statistically significant up-regulated proteins.

Analyzing the up-regulated proteins in the 6D+CBD vs. control MCF-7 cells comparison, it was found that 58 proteins formed a network. As shown in Figure 6, this network contains some of the proteins also present in the Il-1β and CBD networks (Figure 5). It highlights the presence of BRCA1 and MSN and their interaction with the axis formed by SUPT16H, SETD2, and H2BC12. This result shows that 6D cells treated with CBD did not express the same protein levels as MCF-7 cells, although both cells expressed an epithelial phenotype. With regard to the down-regulated proteins in the 6D+CBD vs. MCF-7 cells comparison, three small networks were identified. The first network is formed by the proteins: MAP2K2, YWHAQ, YWHAG, PKN1 and CDK17, the second involves the proteins: ANKRD50, CHD3, MCM5, NCOR2 and RRM1, and the third is formed by: AGR2, AKR1C3, MAGED2 (Supplementary Figure S1C).

## 3. Discussion

Our previous work established that non-invasive breast cancer cells become invasive through the activation of the IL-1β/IL-1RI/β-catenin signaling pathway, leading to epithelial-mesenchymal transition (EMT) [3]. Additionally, it was shown that CBD reversed the EMT by blocking the IL-1β/IL-1RI/β-catenin signaling pathway [8]. And regulation of protein kinase B (AKT) was involved as a crosstalk point [8]. As CBD has been reported to have many cellular effects on cancer progression through various mechanisms of action [50], we performed a proteomic analysis to evaluate the global expression of proteins induced or suppressed by this cannabinoid. In this work, four key aspects are evaluated: (1) identification of proteins that potentially contribute to IL-1β or CBD signaling, (2) discovery of novel crosstalk points between IL-1β and CBD signaling, (3) description of the functions of the main pathways induced by IL-1β and CBD respectively, and (4) characterization of the protein-protein interaction networks in 6D cells treated and not treated with CBD.

### 3.1. Differential Protein Expression Caused by IL-1β or CBD

Our results show that, in 6D cells, five proteins were found present in response to IL-1β stimulation, named by gene symbol: ZNF347, IPO9, SARM1, MTUS2, and TSNAXIP1. All of them are associated with various malignant features in breast cancer. The ZNF347 expression in 6D cells could contribute to the acquisition of invasive features, as this protein has been classified as a predictor of lymph node metastasis from primary breast cancer tumors [18]. Expression of the importin-9 (IPO9) has been implicated in the nuclear transport of F-actin and PFKP proteins, the last one associated with the expression of the CXCL12 chemokine receptor (CXCR4) [19,20]. We have previously demonstrated that IL-1β promotes CXCR4 expression [3]. Thus, it is possible that IPO9 expression mediates the increased levels of CXCR4 and plays a key role in the IL-1β pathway in the 6D cells, regulating β-catenin nuclear translocation. At the same time, IPO9 could control F-actin dynamics in breast cancer cells, as a cancer promoter.

SARM1 regulates the expression of Ki-67 and cyclin D1 (CCND1) [21]. The elevated levels of Ki67 and CCND1, previously observed in 6D cells [7,8], could be due to SARM1 expression. The other IL-1β-induced proteins in 6D cells, MTUS2 and TSNAXIP1, have also been associated with the progression of breast cancer and cytoplasmic remodeling [22,23]. Our findings suggest that these five proteins could serve as potential prognostic markers or therapeutic targets in IL-1β-driven EMT.

Moreover, we found that CBD induced the expression of twelve proteins in 6D+CBD cells, also named here by gene symbol: KRT17, PTMA, RACK1, ABCB4, SCLY, FER, ARFGAP1, SRP19, DNPEP, COG5, DHFR2, and KLHDC7B. The presence of these proteins could promote further mechanisms involved in the reversion or blockage of the malignant changes induced by IL-1β. The induction of keratin 17 (KRT17) has been implicated in the antitumoral IL-17-signaling pathway, and its high expression is reported as a favorable prognosis in breast cancer patients [27]. Although the expression of this keratin is repressed by BRCA1 [51], this datum agrees with our work, as KRT17 was not up-regulated in 6D cells without CBD treatment, which express high levels of BRCA1 (Supplementary Table S1). Therefore, CBD will favor a less aggressive phenotype of 6D cells by enhancing the expression of KRT17. The prothymosin α (PTMA) was also expressed after CBD treatment. This protein can promote estrogen receptor transcriptional activity [28,52] and may have relevance in breast cancer cells. However, further studies are needed to evaluate the role of CBD in the expression of PTMA and the implications of this protein on estrogen receptor activity.

The small ribosomal subunit protein RACK1 enhances the β-catenin stability [31,32]. Therefore, as a protein induced by CBD, we can suggest that its up-regulation could act as a necessary mechanism for restoring the adherens junctions and epithelial morphology by recycling β-catenin from the nuclei in 6D cells. Furthermore, RACK1 is a regulatory cofactor of the phosphatidylcholine translocator ABCB4 [53], which is also induced by CBD (Table 2) and has been reported to suppress colony formation and proliferation of lung cancer cells [33]. In this way, RACK1 and ABCB4 association could suppress the proliferation of the malignant 6D cells by treatment with CBD.

Selenocysteine lyase (SCLY) was also induced by CBD. Its down-regulation has been related to obesity-induced cancers by controlling selenoprotein synthesis [34]. Little is known about this protein, but there is possibly a relation between Se metabolism and CBD signaling. The up-regulation of the tyrosine-protein kinase FER by CBD could directly oppose the formation of F-actin stress fibers, as it has been reported that knock-down of FER results in increased cell spreading and formation of prominent F-actin stress fibers [35]. The ADP ribosylation factor GTPase-activating protein 1 (ARFGAP1) has been reported to inhibit cell growth through mTORC1 signaling and is a prognostic factor of overall survival in pancreatic cancer patients [36]. This result suggests a possible mechanism that is repressing the cell growth of 6D cells via ARFGAP1/mTORC1, explaining the lower viability of 6D cells during the treatment with CBD.

The other proteins, SRP19, DNPEP, COG5, and DHFR2, that were induced by CBD treatment may contribute to the reversal of the malignant features (Table 2). In the literature, SRP19 and DNPEP have been related to p53 activity, inhibition of metastasis, inflammation, and cell proliferation [37,38]; while COG5 mitigates endoplasmic reticulum stress [39] and DHFR2 is considered a favorable prognostic marker in renal cancer [30].

The Kelch domain-containing protein 7B (KLHDC7B) was also induced by CBD. KLHDC7B plays a role in cytoskeletal organization, protein degradation, and gene expression, and has shown clinical utility as a prognostic marker in pan-cancer studies [26]. Furthermore, it has been reported that *KLHDC7B* mRNA is overexpressed in MCF-7 cells [54], and this finding is consistent with our results at the protein level in both control MCF-7 cells and 6D+CBD cells. Furthermore, it was demonstrated that KLHDC7B is induced during the endoplasmic reticulum (ER) stress [55]. And it was also reported that CBD induces ER stress in MCF-7 cells, increasing Ca^2+^ and ROS accumulation [56]. Taken together, this information leads us to hypothesize that KLHDC7B may be up-regulated in CBD-treated cells as a mechanism to response to ER stress induced by CBD. It could serve as a potential marker for the reversion to an epithelial phenotype and could be a novel point of crosstalk between IL-1β and CBD signaling pathways, as IL-1β and CBD induced opposite effects on its expression.

Until now, it is clear that CBD influences the expression of proteins involved in the acquisition of an epithelial phenotype, reversing the aggressive characteristics driven by the IL-1β stimulus. However, CBD may also exert a cytotoxic effect by inducing oxidative stress at both the mitochondrial and endoplasmic reticulum levels [10,57]. Therefore, we propose that, once the malignant phenotype is reversed, CBD may trigger a repair response in the surviving cells that had reverted to an epithelial state from the IL-1β-induced mesenchymal state, alleviating the mitochondrial and ER stress.

### 3.2. Potential Crosstalk Points Between IL-1β and CBD Signaling

Of particular interest in this work was the finding of crosstalk points where the effects of IL-1β and CBD are directly opposite (Table 5). We show now that the expression of KRT16 and RPRD2 corresponded to two new crosstalk points in the pathways involved in the EMT and MET, whose expression was highly up-regulated by IL-1β and then down-regulated by CBD. The up-regulation of KRT16 has been associated with higher tumor aggressiveness, acting as a positive regulator of cellular motility by reorganizing the actin cytoskeleton and in circulating tumor cells of metastatic breast cancer patients, associated with shorter relapse-free survival [58,59]. Therefore, down-regulation of KRT16 by CBD could result in the loss of mesenchymal properties. RPRD2 represses the expression of *miR-205,* which leads to the inhibition of disintegrin and metalloprotease family proteins [60]. While over-expression of *miR-205* promoted the expression of the epithelial marker E-cadherin and reduced other mesenchymal markers [61]. In this work, we found down-regulation of RPRD2 by CBD. Therefore, it can be suggested that in 6D cells treated with CBD, an over-expression of *miR-205* could occur, which agrees with the increased levels of E-cadherin in these cells previously reported and with down-regulation of several mesenchymal markers by CBD [8]. Additionally, 15 other proteins were up-regulated by IL-1β and subsequently down-regulated by CBD, proteins that have been associated with cancer progression and identified as biomarkers of highly aggressive cancer types [49]. Now, we show here that their expression can be down-regulated using CBD.

The Golgi subfamily A member 2 (GOLGA2) was a protein induced by the IL-1β stimulus and then repressed by CBD. This protein regulates cell migration, protein glycosylation, and protein transport across membranes [45]. In addition, its downregulation has been associated with the activation of autophagy, reduction of angiogenesis, and suppression of tumorigenesis in lung cancer [46]. Therefore, we can propose that the down-regulation of GOLGA2 may contribute to the reduction of anticancer features, as observed in our previous findings in 6D tumors treated with CBD [7].

In a similar way, we found 5 proteins that were down-regulated by IL-1β and then up-regulated by CBD (KCNH5, HERC4, MYO1D, EMILIN1, EBF3). The potassium voltage-gated channel KCNH5 is involved in the regulation of cell cycle and proliferation. The up-regulation of the *KCNH5* gene in MCF-7cells is mediated by β estradiol through estrogen receptor alpha (ERα) [62]. Previously, we demonstrated that IL-1β induces up-regulation of TWIS1 that leads to methylation of Erα, decreasing its protein levels [6], suggesting that this mechanism also decreases KCNH5, and after treatment with CBD, it is up-regulated. However, KCNH5 expression and the other four proteins are affected by methylation of their coding gene sequences, and this mechanism seems to be affected by CBD, with implications in tumor development such as cell proliferation, resistance to apoptosis, angiogenesis, invasion, and metastasis. HERC4, a potential E3 ubiquitin-protein ligase, shows contradictory roles, acting as an oncogene or a tumor suppressor [63,64]. We can hypothesize that a potential role of CBD in the inhibition of tumor growth may involve the modulation of ubiquitin ligase activity, including HERC4.

The myosin MYO1D plays a role in endosomal protein trafficking, and especially in the transfer of cargo proteins from the early to the recycling endosome [65]. This protein could be necessary as a mechanism to restore the adherens junctions by recycling the β-catenin protein, as discussed above with RACK1. EMILIN1 is an extracellular matrix protein, and its up-regulation has been associated with a better prognosis of patients with breast cancer. Its knockout, on the other hand, accelerates tumor induction in breast cancer cells [66,67]. The early B-cell factor EBF3 has been found down-regulated in breast cancer tumors and in the malignant MDA-231 cells, and its over-expression leads to the repression of glycolysis [68], an effect that may be attributed to CBD. These findings suggest that these proteins play interconnected roles in breast cancer, with CBD potentially exerting therapeutic effects through multiple mechanisms, all of which are downregulated by IL-1β.

In this work, KRT16 was related to the EMT induced by IL-1β, while KRT17 was linked to the CBD restoration of the epithelial phenotype. Both keratins have been involved with inflammation, differentiation, and proliferation [69]. Using KEGG and GO analysis, we found that 6D cells, with high levels of KRT16, showed up-regulation of pathways involved in the rearrangement of the cytoskeleton, with a clear down-regulation of cadherin binding and cell adhesion pathways (Figure 3). This confirms the phenotypic changes previously observed during the EMT induced by IL-1β [8]. In contrast, in 6D+CBD cells, with high levels of KRT17, down-regulation of the pathways involved in cell migration and cytoskeleton polarity was observed (Figure 4), emphasizing the restoration of epithelial features by CBD.

### 3.3. Protein-Protein Interaction Networks in the IL-1β and in CBD Up-Regulated Proteins

By the proteomic differential expression analysis, we identified a network involving 58 of the IL-1β up-regulated proteins (Figure 5). These proteins are arranged into an axis, comprising three hub proteins: BRCA1, MSN, and CORO1A. The Breast cancer type 1 susceptibility protein (BRCA1) up-regulates Notch signaling, a key event in normal breast tissue differentiation, involving ΔNp63, and JAG1 (Notch ligand) [70]. Previously, we demonstrated that IL-1β induces overexpression of ΔNp63*α* in 6D cells [5]. The increased levels of BRCA1 in these cells could also cause up-regulation of the notch pathway. Moesin (MSN) functions as a cross-linker between cell membranes and actin-based cytoskeletons [71]. Furthermore, MSN interacts with BRCA1, colocalizing with F-actin at the plasma membrane of cancer cells [72]. BRCA1 also negatively regulates the expression of the transcription factors *TWIST* and *C-MYC*, both related to β-catenin nuclear translocation [73]. In our model of 6D cells, the negative effects on the expression of EMT transcription factors by BRCA1 could be suppressed during the MSN-BRCA1 interaction. Additionally, MSN expression correlates with the up-regulation of the *SNAIL1* gene (a key regulator of EMT) and the expression and localization of E-cadherin/β-catenin [74,75]. Previously, we established in the 6D cells that IL-1β induces the up-regulation of *SNAIL1* and promotes the dissociation of adherens junctions [3]. Therefore, the expression of MSN could be another downstream effect of the IL-1β/IL-1RI/β-catenin signaling pathway. Coronin 1A (CORO1A) has been up-regulated in metastatic carcinomas and proposed as a biomarker for metastasis [76,77]. So, it is possible that the axis BRCA1-MSN-CORO1A functions as a complementary signaling pathway that plays a role in driving epithelial-mesenchymal transition (EMT) in response to IL-1β stimulation.

After analysis of the protein network of the up-regulated proteins in 6D+CBD vs. MCF-7 cells comparison, it was clear that both cell types express a differential protein expression, as 6D cells treated with CBD retained some of the proteins induced by IL-1β stimulus, such as BRCA-1- MSN (Figure 6).

On the other hand, the core axis of up-regulated proteins by CBD is formed by SUPT16H, SETD2, and H2BC12. The protein SUPT16H, a component of the facilitated chromatin transcription complex (FACT), is involved in transcription and preservation of chromatin structure [71,78,79]. This protein forms a complex with the proteins SSRP1 and CK2, regulating the P53 activity [80]. SETD2 is a histone methyltransferase that specifically methylates lysine 36 of the histone H3. Methylation of this residue is associated with active chromatin and epigenetic modifications [71,81] Additionally, SETD2 is a tumor suppressor because loss of its function favors cancer progression and chemotherapy resistance by activation of Wnt/β-catenin and ERK pathways [81,82], and its up-regulation has been related with a better prognosis of breast cancer patients [82,83]. Here, we report for the first time that CBD induces up-regulation of the tumor suppressor SETD2 with a possible consequence of reversing the EMT induced by IL-1β.

H2BC12 is a member of the histone H2B family, and its expression is correlated with positive clinical outcomes of glioma patients [71,84]. Therefore, the expression of these three proteins involved in chromatin regulation strongly implies an epigenetic regulation role of CBD in the restoration of an epithelial phenotype. Epigenetic functions of CBD have been described in an encephalomyelitis study where this cannabinoid overwhelms inflammation through histone methylation and ncRNA expression [85]. In this way, CBD could regulate the expression of several proteins involved in P53 signaling, mitochondrial and endoplasmic reticulum stress responses, as well as key regulatory factors of the EMT.

## 4. Materials and Methods

### 4.1. Reagents

Human IL-1β (Peprotech, Rocky Hill, NJ, USA), RH-Oil5 ™ (HempMeds™, Monterrey, NL, Mexico), containing 23.36 mg/mL of purified CBD, was used to prepare a 1000 µM stock solution in DMSO (Sigma-Aldrich, St. Louis, MO, USA).

### 4.2. Cell Culture

The human non-invasive breast cancer cells MCF-7 (ATCC, Manasas, VA, USA) and its derived clone 6D (which is highly responsive to IL-1β stimulus) were used as the cellular model following the previously reported culture conditions [7,8]. The experimental groups included: (1) MCF-7 cells (cells without any treatment were used as a CONTROL), (2) the aggressive 6D cells, and (3) 6D cells treated with CBD (6D+CBD).

All cell cultures were established by seeding 6 × 10^3^ cells/cm^2^ in culture flasks and growing them in DMEM-F12 medium supplemented with 10% fetal bovine serum (FBS), penicillin (5000 U/mL), and streptomycin (5000 µg/mL) from Gibco BRL (Grand Island, NY, USA) at 37 °C with 5% CO_2_ for 48 h. To amplify the homogeneous response of 6D cells to IL-1β stimulation, 20 ng/mL of human IL-1β was added to their growth medium. For 6D+CBD cells, 6D cells grown for 48 h in the presence of IL-1β were rinsed with PBS and incubated in fresh DMEM-F12 medium containing 10 µM of CBD for another 48 h. For all experiments, each type of cell (MCF-7, 6D, and 6D+CBD) was analyzed in triplicate (biological replicates, n = 3).

Before every experiment with 6D cells, features that demonstrated the activation of EMT or MET were evaluated by analyzing changes in cell shape, β-catenin localization, invasiveness, and phosphorylation levels of AKT. The latter was followed to ensure that these transitions occurred in the 6D cells or in the 6D+CBD cells, respectively.

### 4.3. Cell Immunofluorescence

For immunofluorescence assays, MCF-7, 6D, and 6D+CBD cells were first grown on glass coverslips and fixed with 4% paraformaldehyde (PFA) for 20 min and permeabilized with 0.1% Triton X-100 in PBS for 5 min at RT. Next, they were treated with 2% BSA in 0.1% PBS-Tween, and exposed for 1 h at 37 °C to anti-β-catenin antibody (Thermo Scientific, Waltham, MA, USA) (1:100 dilution) and incubated with anti-mouse IgG conjugated with Alexa 488 antibody (1:100 dilution) for 1h at RT. Nuclei were stained with 0.1% DAPI for 5 min. Finally, coverslips were mounted with VectaShield H-1000, and the cells were observed in an Olympus 50× epifluorescence inverted microscope with a digital camera, Olympus DP72. All images were analyzed with Image-Pro Plus software (v. 3.0, Media Cybernetics, Rockville, MD, USA).

### 4.4. Western Blot Assay

Protein extracts were obtained from each type of cell, as previously described [8]. For this, thirty micrograms of protein were loaded per lane and separated by SDS-PAGE in 10% polyacrylamide gels, blotted onto nitrocellulose membranes, and blocked with non-fat milk. The membranes were exposed to the anti-AKT or anti-Phospho-AKT-Ser473 (Cell Signaling Technology, Danvers, MA, USA), rinsed with PBS-Tween, and incubated with HRP-tagged anti-mouse or anti-rabbit secondary antibodies (1:5000) (Jackson Immunoresearch, West Grove, PA, USA). The anti-actin mouse monoclonal antibody, kindly donated by JM Hernández (CINVESTAV-IPN), was utilized to detect actin as a load control. Chemiluminescent detection was carried out with Immobilon™ and recorded on a ChemiDoc imaging device (Bio-Rad Laboratories, Hercules, CA, USA) for densitometric analyses with ImageLab™ software (v 6.0, Bio-Rad Laboratories, CA, USA). Both proteins were identified from the three independent experiments (biological replicates, n = 3).

### 4.5. Invasion Assay

Invasion assays were conducted using 24-well chambers (8 μm pore size) in which the upper side of the Transwell^®^ insert (Corning, Kennebunk, ME, USA) was coated with Matrigel^®^ (Corning, Bedford, MA, USA) for 2 h at 37 °C. For each type of cell, 5 × 10^4^ cells/in 200 µL of DMEM-F12 serum-free medium were placed in the upper chambers, and the lower chambers were filled with 10% FBS supplemented DMEM-F12 medium. After incubation for 24 h at 37 °C under 5% CO_2_ atmosphere, non-invasive cells were removed by scraping with a cotton swab the upper chamber, and the inserts were washed with PBS. The invasive cells at the other side of the filter were fixed with 4% PFA for 15 min and stained with Giemsa for 5 min. Invasive cells were counted under a light microscope at 200× magnification (five fields per insert were counted).

### 4.6. Protein Isolation and Sample Preparation for Mass Spectrometry

Cell monolayers from each condition were washed thrice with PBS and then lysed with 1× RIPA buffer supplemented with Complete™ Protease Inhibitor Cocktail (Roche Applied Science, Mannheim, Germany). Protein concentrations were determined by the BCA method (Pierce™ BCA Protein Assay Kit, Thermo Fisher Scientific, IL, USA).

According to the protocol established by the manufacturer, 50 µg of protein in each sample was cleaned using SP3-iST Kit^®^ (PreOmics, Munich, Germany). Once the samples were cleaned and attached to the magnetic beads, they were enzymatically digested using iST Sample Preparation Kit^®^ (PreOmics) according to the manufacturer and as described by Ortega-Lozano et al. [86], except that we added the “lyse” reagent to the protein attached to magnetic beads instead of a protein pellet.

The resulting peptides were dried using a Savant DNA120 SpeedVac Concentrator (Thermo Fisher Scientific, Waltham, MA, USA), then resuspended in 100 µL of ‘LC-load’ reagent (PreOmics) and sonicated for 5 min in an Ultrasonic Cleaner Bath 2510 (Branson, Brookfield, CT, USA). Finally, samples were stored at −20 °C until mass spectrometry analysis (MS).

### 4.7. Mass Spectrometry-Based Proteomic Analysis

Proteomic analysis was performed in a UPLC Nano Acquity M-Class coupled with a QTOF Mass Spectrometer Synapt G2-S*i* (Waters Corporation; Milford, MA, USA), according to the modified method of Ríos-Castro, et al., 2020 [87]. The spectra data were acquired using a data-independent acquisition (DIA) approach through High-Definition Multiplexed MS/MS (HDMS^E^) mode (Full-Scan DIA). Parameters on the ionization source were set with the following values: 2.75 kV in the capillary emitter, 30 V in the sampling cone, 30 V in the source offset, 70 °C for the source temperature, 0.5 bar for the nanoflow gas, and 150 L·h^−1^ for the purge gas flow. Low and high energy chromatograms were acquired in positive mode in a range of *m*/*z* 50–2000 with a scan time of 500 ms. Precursor ions were fragmented in the transfer cell using a collision energy ramp from 19 to 55 eV [2]. Synapt G2-S*i* was calibrated with [Glu1]-fibrinopeptide, [M+2H]^2+^ = 785.84261 at less than 1 ppm across all tandem mass spectrometry (MS/MS) measurements.

### 4.8. MS-Data Analysis

The MS and MS/MS spectra contained in the generated *.raw files were analyzed by Progenesis QI for Proteomics v4.2 software (Nonlinear Dynamics; Edmonton, AB, CAN) [88,89,90] using a target decoy strategy against a *Homo sapiens* *.fasta database (downloaded from Uniprot on 27 February 2024, UP000005640, 104557 protein sequences) [91,92]. The parameters used for database search were trypsin as cut enzyme and one missed cleavage allowed; carbamidomethyl (C) as a fixed modification and oxidation (M), amidation (C-terminal), deamidation (Q, N) or phosphorylation (S, T, Y) as variable modifications; matching requirements were two fragments per peptide, five fragments per protein, and one peptide per protein [93]; peptide and fragment tolerance were set to automatic and false discovery rate (FDR) ≤ 1%. All false positive identifications (Reversed proteins) and proteins with only one peptide identified were discarded. Proteins were considered differentially expressed if they presented at least a ratio ±1 (expressed as a base 2 logarithm); this means that these proteins had at least ±2-absolute fold change and, *p*-value ≤ 0.05. The ratio was calculated by dividing the average mass spectrometry (MS) signal response of the three most abundant tryptic peptides in the 6D cells by the most abundant tryptic peptides in control MCF-7 cells. In a similar way, the three most abundant tryptic peptides in 6D+CBD cells were divided by the three most abundant tryptic peptides in 6D cells or in MCF-7 cells.

### 4.9. Bioinformatic Analysis

From all the proteins identified in each condition, we removed those marked as “reversed” and retained those with at least two identified peptides per protein (one of which must be unique). To control the probability of false positives, we added a cutoff by measuring the global coefficient of variation (CV) in the three studied comparisons (6D vs. MCF-7, 6D+CBD vs. 6D and 6D+CBD vs. MCF-7), confirming that it was less than 30% in all. A fold change of ±1, expressed as log2, was also applied. Additionally, a False Discovery Rate (FDR) adjustment of the p-value, obtained from a one-way ANOVA test, was performed using the Progenesis QI for Proteomics software. Fold change (expressed as log_2_FC) values between “treated vs. control” conditions were calculated to identify differentially expressed proteins in the cells of the three studied comparisons: “6D vs. MCF-7 cells” and “6D+CBD vs. 6D cells”, and “6D+CBD vs. MCF-7 cells”. Statistically significant proteins were those that passed the two above-described criteria: (1) *p*-value *≤* 0.05 obtained from one-way ANOVA test and (2) a log_2_FC greater than 1 for up-regulated proteins or less than −1 for down-regulated ones. The complete data of analyzed proteins is shown in the Supplementary Tables S1–S3.

To analyze and provide insights on quantitative proteomic data, we used the web-based OmicScope workflow https://omicscope.ib.unicamp.br (accessed on 7 October 2024) to obtain principal components analysis (PCA), volcano plots, and k-clusters [94]. Using PCA of the abundance of each protein revealed distinct clustering of biological replicates and a clear separation between cell types (Supplementary Figure S2A). K-means clustering was used to identify similar expression patterns and establish potential relationships between up-regulated and down-regulated proteins in each cell type (6D vs. MCF-7 and 6D+CBD vs. 6D) (Supplementary Table S4 and Supplementary Figure S2B,C). The SRplot web server “https://www.bioinformatics.com.cn/srplot” (accessed on 14 October 2024) was used to improve the visual quality of volcano plots [95].

Pathway enrichment (KEGG) and Gene Ontology (GO) analyses were performed using ShinyGO™ v0.80 “http://bioinformatics.sdstate.edu/go80/” (accessed on 21 October 2024) on the list of proteins obtained from k-means clustering. These analyses were used to identify biologically overrepresented pathways and to examine the functions of the clustered proteins across three main ontologies: (1) Biological Process (BP), (2) Molecular Function (MF), and (3) Cellular Component (CC) [96].

The networks of predicted associations for differentially expressed proteins, as visualized in the volcano plots, were generated using the Search Tool for the Retrieval of Interacting Genes/Proteins (STRING) version 12.0 “https://string-db.org” (accessed on 9 January 2025). This tool uses experimental and computational data on known and predicted protein-protein interactions, using data obtained from physical and functional interactions to establish protein networks [97].

## 5. Conclusions

In summary, this proteomic study identified proteins expressed in the malignant 6D cells that participate in the IL-1β induction of EMT. In addition, other proteins were identified associated with the reverse process induced by CBD, and 24 new proteins were identified as crosstalk points between IL-1β and CBD signaling pathways. Furthermore, we established that expression levels of some of these proteins could be regulated by one of the two protein axes identified in this work. If the regulation of protein levels is controlled by the axis formed by the proteins BRCA1, MSN, and CORO1A, EMT is active, and the 6D cells remain invasive. In contrast, if the protein regulation is by the axis formed by the proteins SUPT16H, SETD2, and H2BC12, the restoration of epithelial features is activated by CBD, and the cells decrease their invasiveness. Focusing on the main functions of SUPT16H-SETD2-H2BC12 axis, we could hypothesize a potential epigenetic control of CBD on the protein expression. All these results provide new important insights that could help to understand how CBD counteracts the effects of IL-1β and the restoration of the epithelial phenotype as a possible control of cancer progression.

## Figures and Tables

**Table 1:** Proteins detected present or absent in 6D cells.

Uniprot ID	Protein by Gene ID	Description	Role in Cancer Cells
**Present only in malignant 6D cells**			
Q96SE7	ZNF347	Zinc finger protein 347	Predictor of lymph node metastasis [18]
Q96P70	IPO9	Importin-9	Cancer promoter [19,20]
Q6SZW1	SARM1	NAD(+) hydrolase SARM1	Protein involved in maintaining cell viability and proliferation [21]
Q5JR59	MTUS2	Microtubule-associated tumor suppressor candidate 2	Oncogene candidate [22]
B4DXD0	TSNAXIP1	Translin-associated factor X interacting protein 1	Metastasis promoter [23]
**Present in control MCF-7 cells but absent in malignant 6D cells**			
Q8TF65	GIPC2	PDZ domain-containing protein GIPC2	Favorable prognostic marker [24,25]
A0A3B3ISF6	KLHDC7B	Kelch domain containing 7B	Favorable prognostic marker [26]

**Table 2:** Proteins detected present or absent in 6D cells after CBD treatment.

Uniprot ID	Protein by Gene ID	Description	Role in Cancer Cells
**Present only in 6D cells treated with CBD**			
Q04695	KRT17	Keratin_type I cytoskeletal 17	Its decreased expression correlates with poor prognosis [27]
A0A3B3ISF6	KLHDC7B	Kelch domain containing 7B	Favorable prognostic marker [26]
B8ZZA1	PTMA	Prothymosin alpha	Prognostic marker [28,29]
Q86XF0	DHFR2	Dihydrofolate reductase 2_ mitochondrial	Favorable prognostic marker [30]
D6R9L0	RACK1	Small ribosomal subunit protein RACK1	Enhances β-catenin stability [31,32]
P21439	ABCB4	Phosphatidylcholine translocator ABCB4	Its overexpression suppresses colony formation and cell proliferation [33]
H7C1N7	SCLY	Selenocysteine lyase	Control of selenoprotein synthesis [34]
P16591	FER	Tyrosine-protein kinase FER	Its expression correlates with prognosis in breast cancer patients [35]
E5RHC5	ARFGAP1	ADP ribosylation factor GTPase activating protein 1	Inhibits mTORC1 activation [36]
A0A087WYR0	SRP19	Signal recognition particle 19 kDa protein	Regulates p53 activity [37]
B9ZVU2	DNPEP	Aspartyl aminopeptidase	Its overexpression suppresses breast cancer cell proliferation and invasion [38]
Q9UP83	COG5	Conserved oligomeric Golgi complex subunit 5	Mitigates endoplasmic reticulum stress [39]
**Absent in 6D cells treated with CBD**			
A0A5F9ZHY7	BRI3BP	BRI3 binding protein	Decrease apoptosis upon ER stress [40]
K7EM38	ACTG1	Actin_cytoplasmic 2	Tumor promoter [41,42]
O75093	SLIT1	Slit homolog 1 protein	Promotes EMT [43]
O95833	CLIC3	Chloride intracellular channel protein 3	Promotes EMT [44]
A0A6Q8KRG2	GOLGA2	Golgi subfamily A member 2	Tumor promoter [45,46]
Q1KMD3	HNRNPUL2	Heterogeneous nuclear ribonucleoprotein U-like protein 2	Promotes EMT [47]
Q14194-2	CRMP1	Isoform LCRMP-1 of Dihydropyrimidinase-related protein 1	Promotes EMT [48]

**Table 3:** Top ten of significantly up-regulated and down-regulated proteins by IL-1β.

Accession	Protein by Gen ID	Description	Fold Change (6D/MCF-7)	log_2_FC	Anova (*p*)
**Up-regulated in malignant 6D cells**					
P08779	KRT16	Keratin_type I cytoskeletal 16	198.693	7.634	0.001
P23468	PTPRD	Receptor-type tyrosine-protein phosphatase delta	117.515	6.877	0.001
P25815	S100P	Protein S100-P	96.445	6.592	0.026
Q5VT52	RPRD2	Regulation of nuclear pre-mRNA domain-containing protein 2	88.304	6.464	0.007
A0A6Q8KRG2	GOLGA2	Golgin subfamily A member 2	63.978	6.000	0.001
B7ZAA0	PMS1	PMS1 homolog 1_mismatch repair system component	54.681	5.773	0.008
P20807	CAPN3	Calpain-3	46.497	5.539	<0.001
H0Y704	ZNF185	Zinc finger protein 185 with LIM domain	42.648	5.414	0.001
F5H7P7	PPWD1	Peptidylprolyl isomerase	39.739	5.312	0.014
Q8WWI1	LMO7	LIM domain only protein 7	36.108	5.174	0.001
**Down-regulated in malignant 6D cells**					
H3BP20	HEXA	Beta-hexosaminidase	0.04431	−4.496	0.024
A0A0A0MSS8	AKR1C3	Aldo-keto reductase family 1 member C3	0.04954	−4.335	0.003
A0A087X072	RECQL4	DNA helicase	0.06199	−4.013	0.013
O96013	PAK4	Serine/threonine-protein kinase PAK 4	0.09157	−3.449	0.006
H0YGJ7	EIF3J	Eukaryotic translation initiation factor 3 subunit J	0.11865	−3.075	0.005
P02794	FTH1	Ferritin heavy chain	0.16381	−2.610	0.031
P07951	TPM2	Tropomyosin beta chain	0.16528	−2.597	0.033
H0Y626	OX = 9606	B box-type domain-containing protein	0.17925	−2.480	0.001
Q8NCM2	KCNH5	Potassium voltage-gated channel subfamily H member 5	0.18135	−2.463	0.040
Q9H3K2	GHITM	Growth hormone-inducible transmembrane protein	0.19457	−2.362	<0.001

**Table 4:** Top ten of significantly up-regulated and down-regulated proteins by CBD.

Accession	Protein by Gen ID	Description	Fold Change (6D+CBD/6D)	log_2_FC	Anova (*p*)
**Up-regulated in 6D cells by treatment with CBD**					
F8VQD9	ATG101	Autophagy-related protein 101 (Fragment)	897.326	9.809	0.0005
A2PYH4	HFM1	Probable ATP-dependent DNA helicase HFM1	146.677	7.196	0.005
Q5JR59	MTUS2	Microtubule-associated tumor suppressor candidate 2	44.111	5.463	0.03
A0A7P0T9G4	ERCC6	ERCC excision repair 6_ chromatin remodeling factor	26.417	4.723	0.002
Q02386	ZNF45	Zinc finger protein 45	25.611	4.678	0.031
A0A1C7CYW7	TTC34	Tetratricopeptide repeat domain 34	23.524	4.556	0.004
Q92833	JARID2	Protein Jumonji	22.564	4.495	0.023
P54750	PDE1A	Dual specificity calcium/calmodulin-dependent 3′_5′-cyclic nucleotide phosphodiesterase 1A	21.560	4.430	0.0002
Q12901	ZNF155	Zinc finger protein 155	17.148	4.100	0.018
Q12879	GRIN2A	Glutamate receptor ionotropic_ NMDA 2A	16.056	4.005	0.0002
**Down-regulated in 6D cells by treatment with CBD**					
Q9BXL7	CARD11	Caspase recruitment domain-containing protein 11	1.3 × 10^−5^	−16.27	7.8 × 10^−6^
P08779	KRT16	Keratin_type I cytoskeletal 16	1.9 × 10^−2^	−5.69	2.1 × 10^−3^
Q5VT52	RPRD2	Regulation of nuclear pre-mRNA domain-containing protein 2	6.7 × 10^−2^	−3.89	2.2 × 10^−2^
H0YKN8	TLE3	TLE family member 3_transcriptional corepressor	9.2 × 10^−2^	−3.44	1.1 × 10^−3^
Q9GZR1	SENP6	Sentrin-specific protease 6	9.7 × 10^−2^	−3.36	3.2 × 10^−3^
E9PNM1	FDFT1	Squalene synthase	1.0 × 10^−1^	−3.31	4.1 × 10^−2^
Q8WZ60	KLHL6	Kelch-like protein 6	1.2 × 10^−1^	−3.11	4.4 × 10^−2^
P98164	LRP2	Low-density lipoprotein receptor-related protein 2	1.3 × 10^−1^	−2.99	4.6 × 10^−3^
Q53EZ4	CEP55	Centrosomal protein of 55 kDa	1.3 × 10^−1^	−2.93	2.1 × 10^−2^
P41594	GRM5	Metabotropic glutamate receptor 5	1.4 × 10^−1^	−2.87	3.8 × 10^−2^

**Table 5:** Proteins with oppositive expression patterns induced by Il-1β or CBD.

Uniprot ID	Protein by Gene ID	Description	log_2_FC 6D+CBD vs. 6D Cells
**Proteins up-regulated in 6D cells and down-regulated by CBD**			
P08779	KRT16	Keratin_type I cytoskeletal 16	−5.693 ^★^
Q5VT52	RPRD2	Regulation of nuclear pre-mRNA domain-containing protein 2	−3.890 ^★^
H0YKN8	TLE3	TLE family member 3_transcriptional corepressor	−3.440
Q9GZR1	SENP6	Sentrin-specific protease 6	−3.360
P98164	LRP2	Low-density lipoprotein receptor-related protein 2	−2.985
Q4VX76	SYTL3	Synaptotagmin-like protein 3	−1.632
P02545	LMNA	Prelamin-A/C	−1.590
P06702	S100A9	Protein S100-A9	−1.577
Q13509	TUBB3	Tubulin beta-3 chain	−1.573
O15455	TLR3	Toll-like receptor 3	−1.441
Q8NAT2	TDRD5	Tudor domain-containing protein 5	−1.434
Q562F6	SGO2	Shugoshin 2	−1.353
Q8IYB4	PEX5L	PEX5-related protein	−1.165
E9PDI4	LAD1	Ladinin-1	−1.130
P05109	S100A8	Protein S100-A8	−1.124
Q14149	MORC3	MORC family CW-type zinc finger protein 3	−1.088
O14795	UNC13B	Protein unc-13 homolog B	−1.076
**Proteins down-regulated in 6D cells and up-regulated by CBD**			
Q8NCM2	KCNH5	Potassium voltage-gated channel subfamily H member 5	2.887
Q5GLZ8	HERC4	Probable E3 ubiquitin-protein ligase HERC4	1.887
O94832	MYO1D	Unconventional myosin-Id	1.809
Q9Y6C2	EMILIN1	EMILIN-1	1.469
Q9H4W6	EBF3	Transcription factor COE3	1.165
**Protein induced in 6D cells and repressed by CBD**			
A0A6Q8KRG2	GOLGA2 *	Golgi subfamily A member 2	NA
**Protein repressed by IL-1β and induced by CBD**			
A0A3B3ISF6	KLHDC7B ^‡^	Kelch domain containing 7B	NA

* Protein up-regulated in the 6D vs. MCF-7 cells comparison (see Table 3). ^‡^ Protein repressed in the 6D vs. MCF-7 cells comparison (see Table 1). ^★^ Proteins are most significantly down-regulated by CBD.

## Data Availability

The mass spectrometry proteomics data have been deposited to the ProteomeXchange Consortium via the PRIDE [98] partner repository with the dataset identifier PXD059938.

## Supplementary Materials

The supporting information can be downloaded at: https://www.mdpi.com/article/10.3390/ijms26104721/s1.

## Abbreviations

The following abbreviations are used in this manuscript:

CBD	Cannabidiol
EMT	Epithelial-Mesenchymal Transition
MET	Mesenchymal-Epithelial Transition
IL-1β	Interleukin 1 beta
MS	Mass spectrometry
MS/MS	Tandem mass spectrometry
KEGG	Kyoto Encyclopedia of Genes and Genomes
