# Supplementary material for: Proteomic Analysis of Invasive Breast Cancer Cells Treated with CBD Reveals Proteins Associated with the Reversal of Their Epithelial-Mesenchymal Transition Induced by IL-1β
Source: Int J Mol Sci. 2025 May 15;26(10):4721. doi: 10.3390/ijms26104721 (PMC12111826; doi:10.3390/ijms26104721)
Supplement: Supplementary file 1 [file ijms-26-04721-s001.zip › Figure S1.docx]

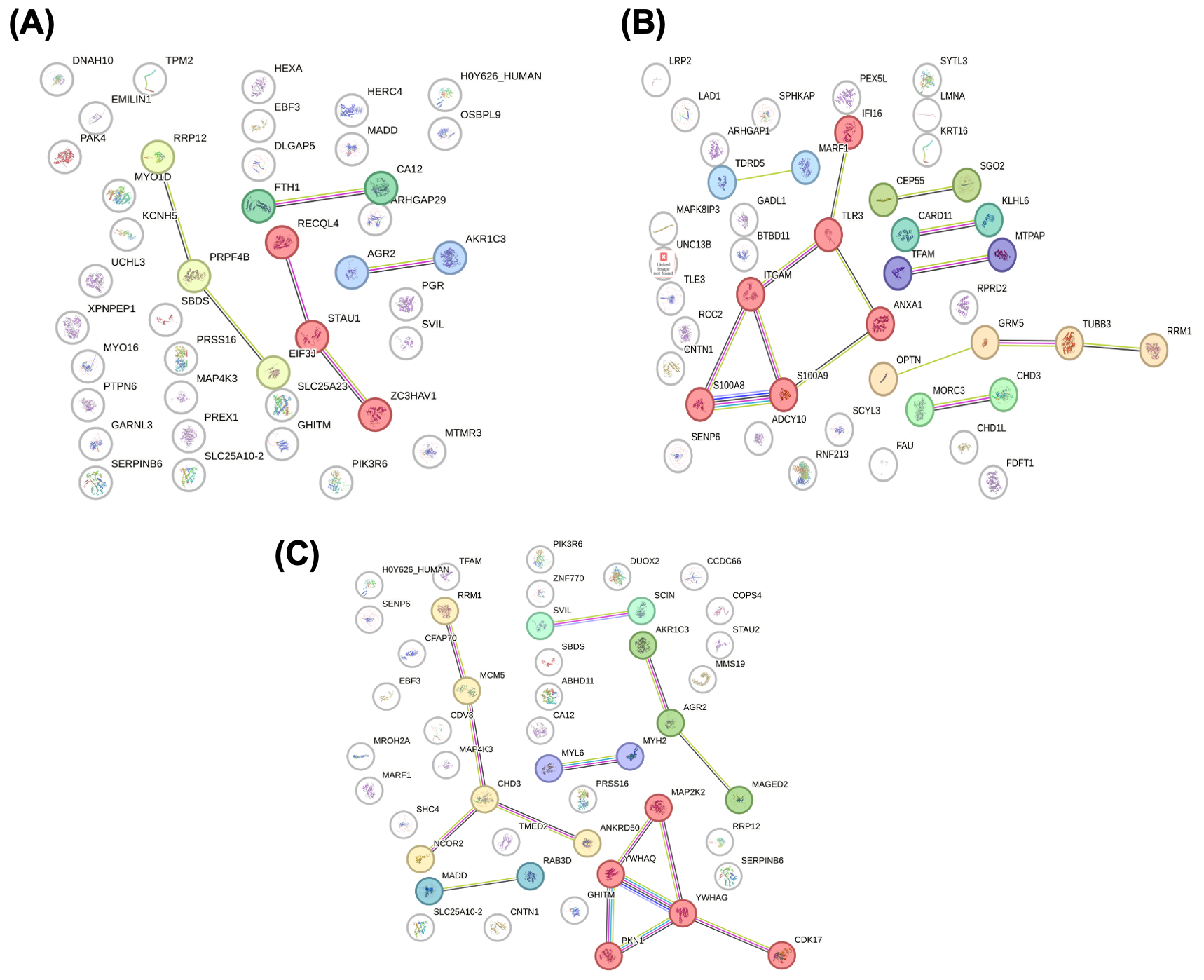


**Supplementary Figure S1.** Protein-protein interaction networks of down-regulated proteins. The interaction analysis was performed with STRING considering a foldchange of log_2_≤﹣1 in down-regulated proteins. The main network is displayed in red in each case. **(A)** 6D vs. MCF-7 comparison. **(B)** 6D+CBD vs. 6D comparison. **(C)** 6D+CBD vs. MCF-7 comparison.
