# Supplementary material for: Proteomic Analysis of Invasive Breast Cancer Cells Treated with CBD Reveals Proteins Associated with the Reversal of Their Epithelial-Mesenchymal Transition Induced by IL-1β
Source: Int J Mol Sci. 2025 May 15;26(10):4721. doi: 10.3390/ijms26104721 (PMC12111826; doi:10.3390/ijms26104721)
Supplement: Supplementary file 1 [file ijms-26-04721-s001.zip › Figure S2.docx]

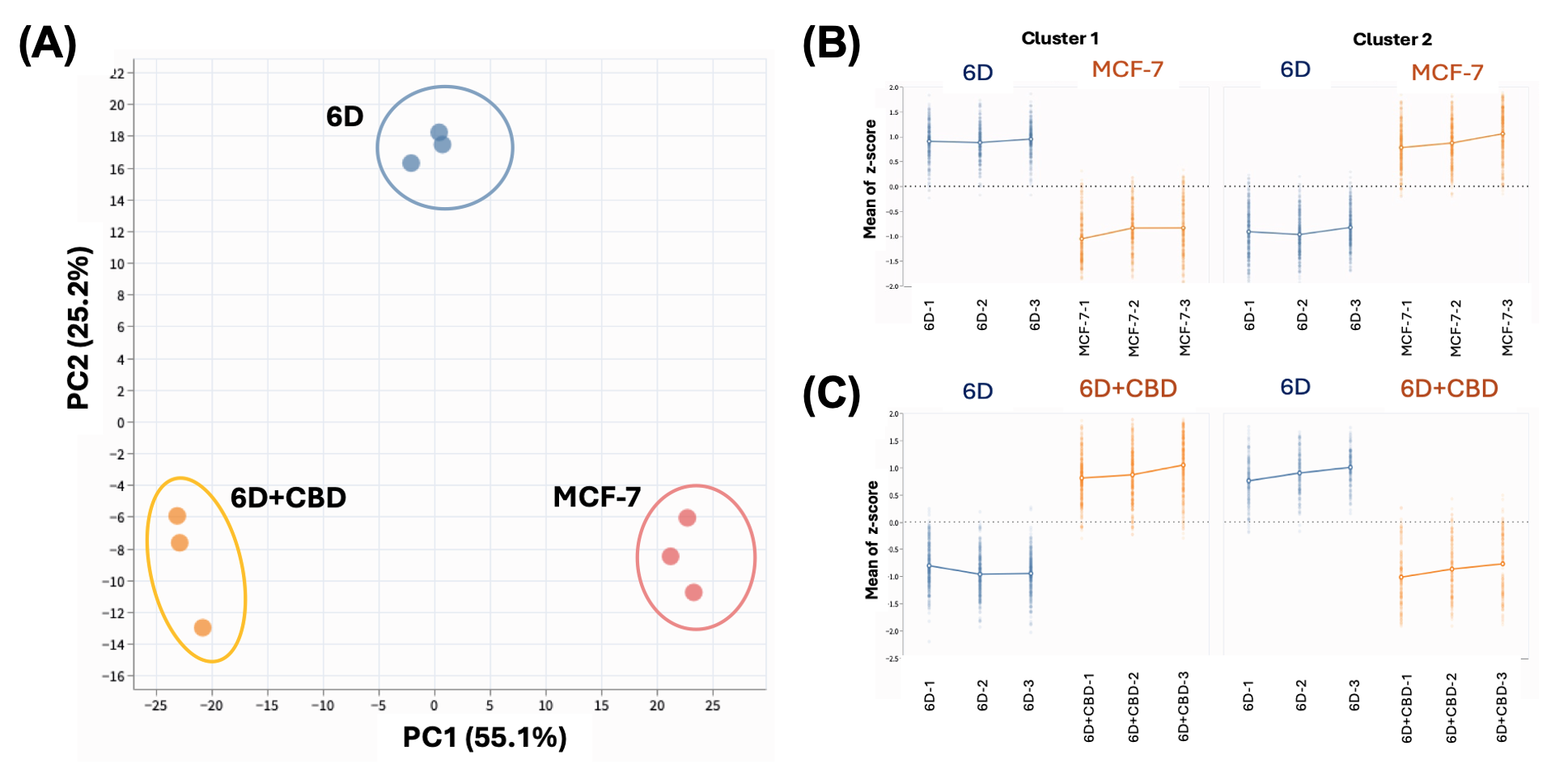


**Supplementary Figure S2.** Principal component analysis and k-means clustering of the protein abundance levels detected in each cell type. **(A)** The biplot displays the clustering analysis based on the first two principal components (PC1 and PC2), which explain the most variance (percentage values are indicated). It reveals that three biological replicates from the same type of cell are grouped closely together, showing a clear distribution of the cell types studied (MCF-7, 6D and 6D+ CBD cells). **(B-C)** Co-expression patterns of proteins expressed in each cell type. The analysis was performed in OmicScope to identify two k-clusters, cluster 1 for up-regulated proteins and cluster 2 for down-regulated in each comparison (6D vs. MCF-7 and 6D+CBD vs. 6D). Three independent biological replicates are shown for each type of cell. The Z-score value indicates how many standard deviations a data point is from the mean of its distribution.
